# Retiring rusty myths around intravenous iron

**DOI:** 10.1002/hem3.70399

**Published:** 2026-06-15

**Authors:** Lauren E. Merz, Arielle L. Langer

**Affiliations:** ^1^ Division of Hematology/Oncology, Department of Medicine University of Michigan Ann Arbor Michigan USA; ^2^ Division of Hematology, Department of Medicine Brigham and Women's Hospital Boston Massachusetts USA; ^3^ Division of Women's Health Brigham and Women's Hospital Boston Massachusetts USA

Iron deficiency anemia affects at least 1.2 billion people worldwide, and the estimates of iron deficiency without anemia are at least twice as high.^1^ Despite the well‐documented excessive burden of disease, iron deficiency is undertreated, and the use of intravenous (IV) iron for treatment is particularly underutilized in high‐resource settings. We hope to reduce barriers to IV iron use by identifying and dispelling some common myths.

## MYTH: IRON DEFICIENCY WITHOUT ANEMIA DOES NOT REQUIRE TREATMENT

Too often, iron deficiency is not considered or screened for until anemia is detected. However, anemia is a late consequence of iron deficiency, and there are many meaningful consequences of iron deficiency that warrant treatment even in the absence of anemia.^1^ For example, restless legs can be ameliorated with iron supplementation, which can be as effective as other pharmacologic approaches without long‐term side effects.^2^ Exercise capacity is reduced in those with iron deficiency without anemia and normalized after treatment.^1^ Among adolescents with iron deficiency without anemia, cognitive scores are improved with iron supplementation and resolution of iron deficiency.^3^ Maternal and fetal outcomes are worse among those who are iron‐deficient, including worse language and motor outcomes at 2 years old.^4^ Furthermore, iron deficiency is independently associated with decreased quality of life and increased all‐cause mortality.^5^ Thus, identifying and treating iron deficiency with or without anemia is necessary and highly beneficial.

## MYTH: ORAL IRON IS JUST AS EFFECTIVE AS IV IRON

While oral iron is appropriate in many cases, there are situations in which IV iron would be preferred. There are four major considerations: the timeframe for improvement, the degree of ongoing deficits, intolerance to oral iron, and the expected degree of absorption. For example, patients with upcoming surgeries or pregnant patients in the second or third trimester should be given IV iron for rapid correction. Patients with any history of intolerance to oral iron due to constipation, dyspepsia, or nausea should be given IV iron. This is especially critical to consider in elderly patients, where prescription cascades from iron to antacids, and iron to laxatives are common.^6^ In patients with any difficulty in absorbing oral iron from malabsorptive states like celiac disease or bariatric surgery or from high levels of inflammation, such as congestive heart failure or chronic kidney disease, oral iron administration is predictably futile.^1^ While many patients are well served by oral iron, using it as the default first‐line approach or continuing chronic use despite intolerance or failure to correct overlooks situations in which it will clearly be ineffective.

## MYTH: INTRAVENOUS IRON IS TOO EXPENSIVE FOR COMMON USE

IV iron itself is more expensive than oral iron, but this is too simplistic and fundamentally miscalculates the true costs. Timely use of IV iron can mitigate other high‐cost medical care, such as transfusion or hospitalization.^7^ More importantly, the appropriate metric for understanding costs should not be limited to the price tag of each medication but should be calculated as an incremental cost‐effectiveness ratio (ICER), which accounts for associated appointments, laboratory studies, quality‐of‐life impacts, and time missed from the labor force. For example, the ICER for first‐line IV iron in patients with heavy menstrual bleeding is $28,600 per quality‐adjusted life year (QALY).^8^ This is well within the ICER used by leading bodies such as the United Kingdom's National Institute for Health and Clinical Excellence (NICE) to define cost‐effective care. While additional research to calculate the ICER in other clinical contexts is needed, the premise that IV iron is too expensive inherently fails to account for the costs of avoiding its use and should be abandoned in favor of more holistic and rigorous analysis.

## MYTH: WE SHOULD NOT OFFER INTRAVENOUS IRON BECAUSE IT IS TOO HARD TO GET PATIENTS THE INFUSION

Many general practitioners lack privileges at infusion centers that administer IV iron or are not comfortable writing infusion orders. Additionally, wait times and limited access to hematologists are a common reality in many healthcare systems. Although these are barriers, they should be rejected as the status quo. Rather, an intentional, systems‐based approach is needed to ensure timely access to infusion spaces and to increase providers' confidence in appropriately prescribing IV iron. There are many examples of effective system‐level changes to optimize efficiency, such as dedicated infusion chairs for IV iron, provider education or decision‐support tools to facilitate appropriate ordering, and prioritization of rapid‐infusion and single‐dose formulations.^9^

## MYTH: INTRAVENOUS IRON CAUSES FREQUENT AND DANGEROUS INFUSION REACTIONS

Anaphylaxis is a real complication of IV iron infusion, but incidence rates are incredibly low and reported from 0.8 to 9.8 per 10,000 depending on the formulation.^10^ This is similar to incidence rates for anaphylaxis to beta‐lactam antibiotics. Furthermore, these IV iron rates are likely overestimates as Fishbane reactions are often mistaken for anaphylaxis. A Fishbane reaction is a complement activation–related pseudoallergy that occurs in 1%–3% of IV iron infusions, usually within the first few minutes of administration. Symptoms can include flushing, back or chest pressure, anxiety, mild dyspnea, mild tachycardia, or mild hypotension. However, there is no urticaria, bronchospasm, or vascular collapse. Symptoms resolve within 5–10 minutes of stopping the infusion, do not require additional intervention, and usually permit resumption of infusion. Although patients should be counseled about the possibility of uncomfortable Fishbane reactions, these must be distinguished from a true anaphylactic reaction and should not discourage continued administration of this vital medication.

## MYTH: INTRAVENOUS IRON IS DANGEROUS IF A PATIENT HAS AN INFECTION

IV iron is often not given during inpatient admission due to concerns about the therapy worsening the infection. However, there are very few siderophilic bacteria, and the most common ones are relatively rare causes of infection like *Vibrio* and *Yersinia*. A recent real‐world study of iron‐deficient patients with MRSA bacteremia, pneumonia, urinary tract infection, colitis, and cellulitis found that IV iron use significantly reduced mortality at 14 and 90 days compared to those without IV iron administration.^11^ IV iron use was also unsurprisingly associated with increases in hemoglobin from baseline.^11^ These data suggest that we may be vastly overestimating the risks of IV iron in patients with systemic and localized infections as well as neglecting an opportunity to improve outcomes.

In summary, limited IV iron use may stem from myths and misconceptions around the need for treatment, access to treatment, and risks associated with administration. The key to myth‐busting often lies in education efforts. However, system‐level interventions, including utilizing electronic medical records to provide decision aids around IV versus oral iron, access to infusion centers, training to increase confidence in ordering therapy, and prioritizing single‐dose and rapid‐infusion protocols, can also facilitate increased utilization of IV iron. Iron deficiency is highly prevalent and highly treatable, and IV iron is an effective and underutilized therapy to combat this epidemic.

## CONFLICT OF INTEREST STATEMENT

The authors declare no conflicts of interest.

## FUNDING

This research received no funding.

## Data Availability

Data sharing is not applicable to this article as no datasets were generated or analyzed during the current study.
